# Association between a combined lifestyle exposure index and age at pancreatic cancer diagnosis: a hospital-based retrospective study

**DOI:** 10.1186/s12876-026-05297-0

**Published:** 2026-09-10

**Authors:** Fu-Jen Lee, Lung-Chang Chien, Jaw-Town Lin, Chun-Chieh Yeh, Ro-Ting Lin

**Affiliations:** 1 Graduate Institute of Public Health, College of Public Health, China Medical University, Taichung, 406040 Taiwan; 2 Division of Gastroenterology and Hepatology, Department of Internal Medicine, Fu Jen Catholic University Hospital, New Taipei City, 24352 Taiwan; 3 Department of Epidemiology and Biostatistics, School of Public Health, University of Nevada, Las Vegas, Las Vegas, NV 89154 USA; 4 Division of Gastroenterology and Hepatology, Department of Internal Medicine, E-Da Hospital, Kaohsiung, 824005 Taiwan; 5 Department of Surgery, China Medical University Hospital, Taichung, 404332 Taiwan; 6 Department of Occupational Safety and Health, College of Public Health, China Medical University, Taichung, 406040 Taiwan

**Keywords:** Pancreatic cancer, Age at diagnosis, Combined exposure index, Lifestyle risk factors, Smoking, Diet, Retrospective study

## Abstract

**Background:**

Pancreatic cancer is a highly lethal malignancy, and its incidence is increasing among younger individuals. Although smoking, alcohol use, and unhealthy diet are established risk factors, the cumulative impact of multiple lifestyle exposures on age at diagnosis remains unclear. This study aimed to estimate the combined burden of lifestyle and environmental risks associated with the timing of pancreatic cancer diagnosis.

**Methods:**

We conducted a hospital-based retrospective case-only study including 134 patients diagnosed with pancreatic cancer between 2020 and 2025. Data on family history of pancreatic cancer, cigarette smoking, alcohol consumption, dietary patterns, exercise, and occupational chemical exposure were collected through structured interviews and medical record reviews. Cox proportional hazards models using age at diagnosis as the time scale were used to evaluate associations between exposures and earlier diagnosis. Lifestyle factors were integrated into a cumulative exposure index and analyzed using Cox models with adjustment for the remaining covariates.

**Results:**

The mean age at diagnosis was 64.8 years. A significant difference in age at diagnosis was observed across smoking categories, with current smokers having the youngest mean age, whereas no significant difference was observed across alcohol consumption categories. Patients with triple exposure had the youngest mean age at diagnosis (55.5 years), followed by those with double (63.5 years), single (64.8 years), and no (69.0 years) exposures. Compared with no exposure, single, double, and triple exposures were associated with progressively higher hazards of earlier diagnosis (HR = 1.79, 95% CI: 1.14–2.81, *p* = 0.011; HR = 2.97, 95% CI: 1.67–5.28, *p* < 0.001; and HR = 6.66, 95% CI: 3.35–13.22, *p* < 0.001, respectively).

**Conclusions:**

Our findings indicate a significant association between a higher combined exposure index and earlier age at pancreatic cancer diagnosis. This exposure–response gradient suggests that a greater accumulation of adverse lifestyle exposures corresponds to earlier age at diagnosis among affected patients.

**Trial registration:**

Not applicable.

**Supplementary Information:**

The online version contains supplementary material available at https://doi.org/10.1186/s12876-026-05297-0.

## Background

Cancer remains a major global health burden, with its incidence increasing sharply with age. However, several malignancies are increasingly being diagnosed in younger individuals [1–3]. This emerging trend has raised concerns that multiple exposures may increase cancer risk and accelerate disease onset [2, 4, 5], potentially through immune dysregulation or biological aging [6, 7]. Established carcinogens, such as cigarette smoking and alcohol consumption, illustrate this concept: their co-occurrence has been linked to higher cancer risk, suggesting that the combined burden of exposure may contribute to earlier diagnoses [8, 9].

Pancreatic cancer exemplifies this concern owing to its poor prognosis and rising incidence among younger individuals [10–12]. Most patients are diagnosed at an advanced or metastatic stage, when curative treatment is rarely feasible, highlighting the critical need for earlier detection and improved identification of high-risk individuals [13, 14]. A broad range of risk factors have been implicated, including genetic susceptibility, lifestyle behaviors, metabolic conditions, and occupational or environmental exposures [15–17]. Despite this diversity, most single exposures confer only modest relative risks, and the determinants of earlier disease onset remain poorly characterized. Previous studies have indicated that smokers and heavy alcohol consumers are diagnosed 4–8 years earlier than unexposed individuals [18–26]. However, these studies have largely examined individual exposures, with few addressing the cumulative effect of multiple co-occurring risk factors on the age at diagnosis.

To address this gap, we established a hospital-based retrospective case-only study to investigate whether combined exposure is associated with earlier pancreatic cancer diagnosis [27]. Our prior analysis found that smokers and drinkers were diagnosed approximately six years earlier than non-users, and individuals with both exposures were diagnosed nearly a decade earlier [28]. Although these findings suggest that multiple harmful exposures may be associated with earlier diagnosis, they were limited to two lifestyle factors and relied on comparisons of the mean age. The contribution of a broader set of exposures to earlier diagnosis remains unclear.

The present study expands on this work by systematically evaluating the cumulative impact of a wider range of exposures, including dietary behaviors, physical activity, chemical exposure, traditional lifestyle factors (smoking and alcohol consumption), and familial risk factors. We aimed to investigate whether the accumulation of multiple adverse exposures is associated with a younger age at pancreatic cancer diagnosis and to provide a comprehensive framework for understanding how co-occurring risk factors may influence disease timing, thereby addressing a critical gap in current pancreatic cancer research.

## Methods

### Study design and participants

This hospital-based retrospective case-only study was conducted at China Medical University Hospital (CMUH), a tertiary medical center in central Taiwan. This observational study was designed and reported in accordance with the STROBE (Strengthening the Reporting of Observational Studies in Epidemiology) guidelines to support transparent and complete reporting of observational research; a completed STROBE checklist is provided as Additional file 1. The study design and recruitment procedures followed those of our previous investigations of pancreatic cancer risk factors in the same clinical setting (IRB numbers: CMUH109-REC3-026, CMUH111-REC3-084, and CMUH113-REC3-045) [27, 28]. Eligible participants were patients with a confirmed diagnosis of pancreatic cancer (ICD-10 codes C25.0–C25.9) who were admitted between July 1, 2020, and November 30, 2025. Patients were recruited from the Departments of Gastroenterology, Hematology, Oncology, and Surgery. Written informed consent was obtained from all participants prior to enrollment. The final analytic sample included patients with complete questionnaire and medical record data; individuals with incomplete questionnaire data were excluded before analysis. No formal sample size calculation was performed. The study size was determined by the number of eligible, consenting patients with complete questionnaire data available during the recruitment period.

### Setting

Data collection took place during hospital admission and involved structured face-to-face interviews supplemented by a review of medical records. The total study period spanned 5 years.

### Data collection and variable definitions

Demographic and epidemiological data were collected through structured face-to-face interviews conducted during hospitalization and supplemented by a review of medical records during hospitalization. Interviews were administered by trained research staff using a standardized questionnaire to ensure consistent data collection across participants. Variables included age, sex, education, family history of pancreatic cancer, cigarette smoking, alcohol consumption, regular exercise, dietary habits, and chemical exposure. Clinical information such as diagnosis and relevant medical history was verified through medical record review when available.

Several procedures were used to reduce potential sources of bias. Standardized face-to-face interviews administered by trained research staff were used to reduce interviewer variability, medical records were reviewed to verify diagnoses and clinical information when available, and exposure definitions were harmonized before analysis. Because lifestyle, dietary, and occupational exposures were self-reported retrospectively, recall bias and measurement error remained possible and are addressed in the Discussion.

Education was categorized according to the highest attained level (elementary or lower, junior/high school, or college or higher). In Taiwan, formal education typically consists of 6 years of primary school, 9 years including junior high school, 12 years including senior high school, and 14–16 years for college or university education. These values therefore reflect cumulative years of schooling based on standard educational milestones. Accordingly, for continuous analyses, years of schooling were assigned as 6, 9, 12, 14, 16, 18, or 22, corresponding to increasing educational levels. A family history of pancreatic cancer was defined as having at least one first-degree relative diagnosed with the disease.

Cigarette smoking was defined as having smoked more than 100 cigarettes (approximately five packs) over a lifetime. Participants were classified as smokers if they were current smokers or had quit smoking within the past 10 years. Smoking status was categorized as never, current, or former smoking based on participants’ self-reported history. For the combined exposure index, cigarette smoking was coded as a binary variable in which both current and former smokers were classified as exposed and never smokers as unexposed.

Regarding alcohol consumption, participants were asked about the types of alcoholic beverages typically consumed (e.g., beer, wine, or spirits) and the approximate amount consumed. Reported amounts were converted into an approximate beer-equivalent volume based on typical serving sizes to facilitate comparison across beverage types. Habitual alcohol consumption was defined as intake exceeding approximately 350 mL per day within the six months prior to diagnosis, which corresponds roughly to one standard can of beer and facilitates recall during interviews. Alcohol use was initially categorized as never, current, or former drinking status. For the combined exposure index, alcohol consumption was coded as a binary variable in which current and former drinkers were classified as exposed and never drinkers as unexposed.

Regular exercise was measured as a lifetime time-weighted average from age 15 to the current age, with values ranging from 0 (never), 0.5 (intermittent), to 1 (regular annual exercise).

Dietary exposure was assessed using 10 variables based on a 2-year recall, including weekly frequency of Chinese medicine use, fatty meats, pork belly, animal skin, viscera, fried chicken, French fries, egg yolk, dessert, and sugary drinks. These variables were selected based on the study questionnaire and prior literature review, focusing on commonly consumed food items with potential adverse health effects [29–32]. Dietary intake was recorded as frequency of consumption and harmonized to weekly servings. Missing component values were imputed before percentile normalization, and each variable was converted to a percentile scale (0–100), representing relative consumption patterns rather than absolute portion sizes. Higher scores indicated a greater intake of high-fat, high-sugar foods, and more frequent use of Chinese medicine. Chemical exposures were assessed via participant reports of occupational or environmental contact with substances such as organic solvents, dust, adhesives, degreasers, resins, paints, formaldehyde, and other chemicals. Exposure to one or more of these categories was recorded as chemical exposure (binary coded: 1 = yes, 0 = no).

### Outcome measure

The primary outcome was age at pancreatic cancer diagnosis, extracted from hospital medical records and measured in years. This continuous variable was used in both descriptive and time-to-event analyses. Because all participants were pancreatic cancer cases, the study did not assess pancreatic cancer incidence or disease risk in the source population.

### Statistical analysis

Patient characteristics were summarized using frequencies and percentages for categorical variables and means, standard deviations, and medians for continuous variables. Age at pancreatic cancer diagnosis was compared across exposure groups using the Mann–Whitney U test for binary variables and the Kruskal–Wallis test for variables with three or more categories.

To evaluate the cumulative effects of multiple correlated exposures, such as the diet and chemical indices, we applied a grouped weighted quantile sum (WQS) regression to derive composite exposure indices for risk factors [33]. Ten diet-related variables and 10 chemical exposure variables were grouped into two mixtures (diet and chemical). Each exposure was transformed into tertile ranks, and within-group weights were derived using 500 bootstrap samples constrained to be non-negative and summed to 1. The resulting indices were then entered simultaneously into a linear model, with age at pancreatic cancer diagnosis as the outcome. Covariates included sex, years of education, family history of pancreatic cancer, smoking status, alcohol use, and average lifetime exercise.

Next, we fitted a Cox proportional hazards model including all candidate exposure indices simultaneously to identify lifestyle factors associated with age at diagnosis. Age at diagnosis was treated as the time-to-event outcome, with age serving as the underlying time scale (time since birth). In this framework, all individuals in the study experienced the event (pancreatic cancer diagnosis), and therefore no censoring occurred. Hazard ratios derived from these Cox models should therefore be interpreted as relative differences in the timing of diagnosis among pancreatic cancer cases rather than differences in disease incidence, and should not be interpreted as causal effects on disease occurrence.

We first fitted a multivariable Cox model including all candidate exposure indices simultaneously to identify lifestyle factors associated with age at diagnosis, adjusting for core covariates:

Model 1 $$\begin{array}{ll} h(t) &= h_{0}(t)\exp(\beta_{1}[\mathrm{sex}] +\beta_{2}[\mathrm{education}] +\beta_{3}[\text{family history}] \\ &\qquad +\beta_{4}[\mathrm{exercise}] +\beta_{5}[\text{cigarette smoking}] \\ &\qquad+\beta_{6}[\text{alcohol consumption}] +\beta_{7}[\text{diet index}] \\ &\qquad+\beta_{8}[\text{chemical index}]). \end{array}$$

where $\:h\left(t\right)$ is the hazard function for age at pancreatic cancer diagnosis at time * t*, with * t* representing years from birth until diagnosis, and $\:{h}_{0}\left(t\right)$ denoting the baseline hazard function. The parameters $\:{\beta\:}_{i}$ represent the regression slope for each variable.

Based on Model 1, cigarette smoking and dietary index were significantly associated with earlier diagnosis. Alcohol consumption showed a similar direction of association but did not reach statistical significance. Because the combined exposure index was designed to capture the accumulation of adverse lifestyle exposures, cigarette smoking, alcohol consumption, and dietary index were retained as the lifestyle factors of interest to construct the combined exposure index. In contrast, variables such as exercise intensity were treated as covariates rather than components of the exposure index because they represent protective behaviors rather than adverse exposures. The combined exposure index was the sum of three binary indicators: cigarette smoking, alcohol consumption, and dietary index (coded 1 = yes, 0 = no; dietary index was defined as diet index > cohort median). The combined exposure index ranged from 0 to 3, categorized as 0 = none, 1 = single exposure, 2 = double exposure, and 3 = triple exposure. This categorization was partly inspired by the counting framework of Fukai et al. (2020) [8]. This approach was chosen to provide a simple and interpretable measure of cumulative exposure burden while minimizing model complexity given the sample size. Because the index was constructed post hoc and discretized, it was interpreted as an exposure-count index rather than a biologically calibrated continuous dose gradient. We thus fit Model 2:

Model 2 $$\begin{array}{l} h(t)=h_{0}(t)\exp(\beta_{1}[\mathrm{sex}] +\beta_{2}[\mathrm{education}] +\beta_{3}[\text{family history}] \\ \qquad +\, \beta_{4}[\mathrm{exercise}] +\beta_{5}[\text{chemical index}]\\\qquad +\, \beta_{6}[\text{combined exposure index}]) \end{array}$$

Each estimated coefficient $\:\widehat{\beta\:}$ was exponentiated to obtain a hazard ratio (HR), indicating the relative likelihood of earlier pancreatic cancer diagnosis associated with each factor among cases. The proportional hazards assumption was evaluated using Schoenfeld residual-based diagnostics with 5,000 resampling iterations and a fixed random seed. Sensitivity analyses were conducted using reduced Cox proportional hazards models in which each covariate was sequentially removed to evaluate the stability of the combined exposure index estimates. As an additional sensitivity analysis, tumor stage at diagnosis was incorporated into the Cox models among participants with available stage information. Stage was classified into ordinal categories (I, II, III, IV) reflecting increasing disease severity and included as a categorical variable to evaluate whether the observed associations were influenced by stage at diagnosis. All analyses were conducted using SAS version 9.4 (SAS Institute, Cary, NC), with a two-sided significance level of 0.05.

## Results

The final analytic sample comprised 134 patients with complete questionnaire data and confirmed pancreatic cancer; a participant flow chart is provided as Additional file 2**.** Table 1 summarizes the characteristics of the 134 participants. The mean age at diagnosis was 64.8 years (SD = 11.2). Significant differences were observed in the educational and lifestyle factors. Patients with ≥ 12 years of education were diagnosed at a younger age (60.0 years) compared to those with < 12 years (69.1 years; *p* < 0.001). A significant difference in age at diagnosis was observed across smoking categories (*p* = 0.004), with current smokers having the youngest mean age. No significant difference was observed across alcohol consumption categories (*p* = 0.074). A similar pattern was observed for diet: participants above the cohort median were diagnosed earlier than those below (60.4 vs. 69.1 years; *p* < 0.001).

**Table 1:** Descriptive characteristics of study participants (*N* = 134). Age at diagnosis is reported in years

Variables	*N*	%	Mean age at diagnosis (years)	Standard deviation (years)	*p* value ^e^
Overall	134	100	64.76	11.26	-
ICD-10 diagnosis					
C25.0	82	61	65.26	10.86	0.441
C25.1	23	17	66.33	12.64	
C25.2	25	19	62.08	10.01	
C25.9	4	3	62.13	19.18	
Sex					
Male	77	57	65.20	10.73	0.732
Female	57	43	64.17	12.01	
Educational years					
< 12	70	52	69.10	8.51	< 0.001
≥ 12	64	48	60.01	12.02	
Family history of pancreatic cancer					
No	129	96	64.66	11.43	0.690
Yes	5	4	67.28	5.05	
Average exercise intensity ^a^					
Lower level	77	57	64.55	10.09	0.386
Higher level	57	43	65.04	12.76	
Cigarette smoking					
Never	82	61	65.85	11.66	0.004
Former	28	21	67.35	7.78	
Current	24	18	58.02	11.10	
Alcohol consumption					
Never	102	76	65.76	11.19	0.074
Former	18	13	63.46	11.19	
Current	14	11	59.13	10.80	
Diet index ^b^					
Lower level	67	50	69.14	8.84	< 0.001
Higher level	67	50	60.38	11.76	
Chemical index ^c^					
Lower level	67	50	62.90	12.86	0.069
Higher level	67	50	66.62	9.11	
Combined exposure index ^d^					
0 (none)	40	30	69.04	9.98	< 0.001
1 (single exposure)	52	39	64.81	11.90	
2 (double exposure)	27	20	63.48	8.72	
3 (triple exposure)	15	11	55.47	11.06	

Abbreviations: ICD-10 = International Statistical Classification of Diseases and Related Health Problems, 10th Revision; N = number of participants; WQS = weighted quantile sum

^a^ Average exercise intensity: lifetime time-weighted average exercise score (0 = never, 0.5 = intermittent, 1 = regular), categorized into lower and higher levels by the median

^b^ Diet index: composite dietary exposure factor derived using the grouped weighted quantile sum (WQS) regression, categorized into lower and higher levels by the median

^c^ Chemical exposure index: composite chemical exposure factor derived using the grouped WQS regression, categorized into lower and higher levels by the median

^d^ Combined exposure index was constructed as the sum of three binary indicators—cigarette smoking (current or former = 1, never = 0), alcohol consumption (current or former = 1, never = 0), and dietary pattern (above the cohort median = 1, at or below = 0). The index ranged from 0 to 3, with 0 = none, 1 = single, 2 = double, and 3 = triple exposure. In the Cox proportional hazards models, this index was entered as a categorical variable to estimate the association between cumulative adverse lifestyle exposures and earlier age at diagnosis among pancreatic cancer cases

^e^ Differences in mean age between two groups were assessed using the Mann-Whitney U test, and differences among three or more groups were assessed using the Kruskal-Wallis test

When exposures were combined into an index, mean age at diagnosis decreased progressively with increasing exposure count: no exposure (69.0 years), single exposure (64.8 years), double exposure (63.5 years), and triple exposure (55.5 years; *p* < 0.001), indicating a stepwise association between higher exposure count and younger age at diagnosis. In contrast, no significant differences in mean age were observed according to ICD-10 diagnosis, sex, family history, exercise intensity, or chemical exposure index.

We constructed a multi-index model incorporating two composite indices derived from the grouped WQS method: a dietary exposure index and a chemical exposure index. As shown in Figure **S1** (see Additional file 3), the dietary index was dominated by fried chicken (69%), French fries (18%), and sugar-sweetened beverages (9%), which accounted for approximately 96% of the index. The chemical index was primarily driven by other chemicals (54%) and pesticides (14%), followed by dry-cleaning exposure (10%) and asphalt (10%), cumulatively representing approximately 88% of the index. The remaining components, such as desserts, viscera, incense, and metals, contributed only marginally.

**Fig. 1:**
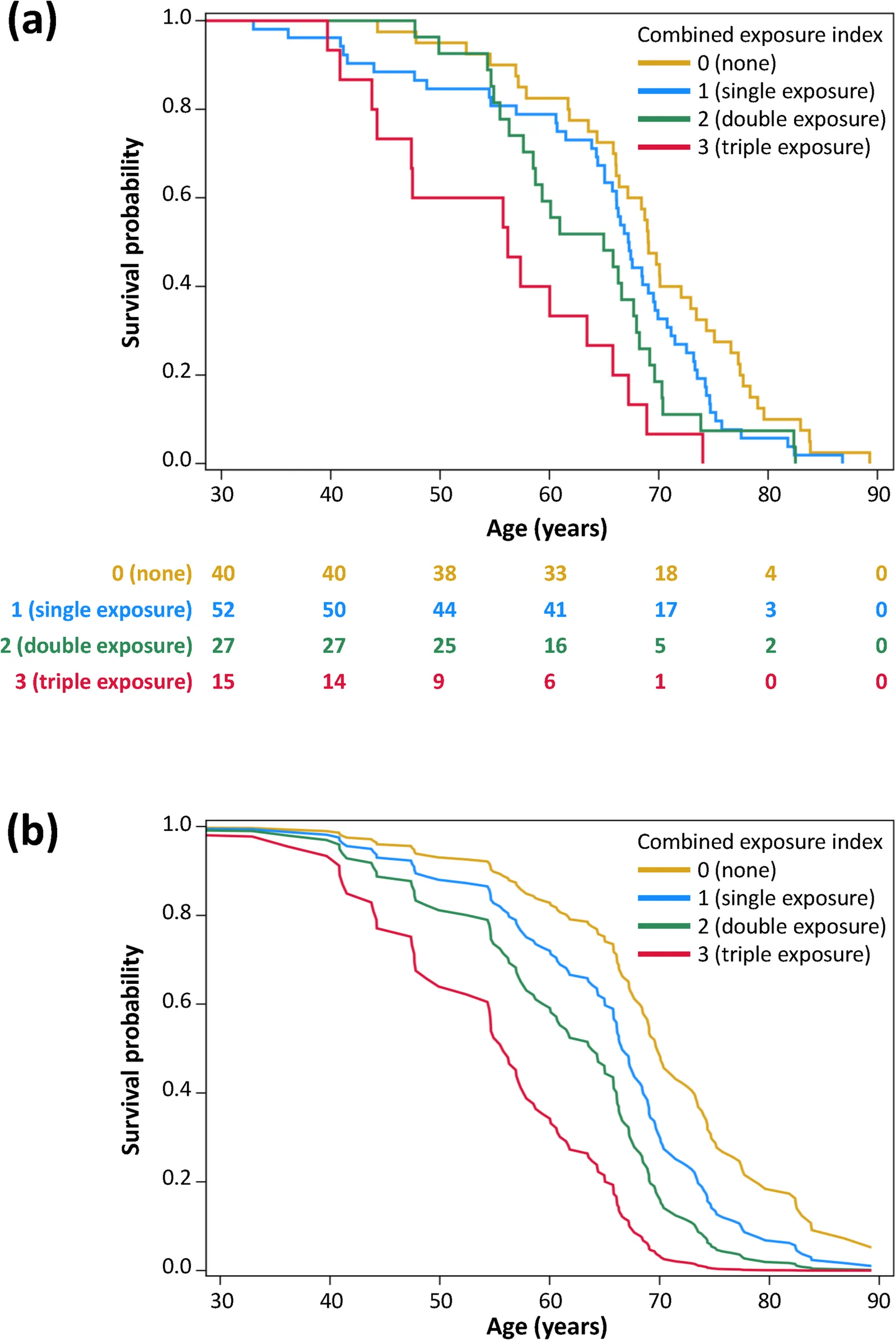
Age-at-diagnosis functions by the combined exposure index. **a** Kaplan-Meier survival curves showing the probability of not yet being diagnosed with pancreatic cancer by age (years) among pancreatic cancer cases, stratified by the combined exposure index (0 = none, 1 = single exposure, 2 = double exposure, 3 = triple exposure). **b** Adjusted survival functions estimated from the Cox proportional hazards model with age at diagnosis as the time scale, controlling for sex, education, family history, physical activity, and chemical exposure. The combined exposure index was the sum of three binary indicators: cigarette smoking, alcohol consumption, and dietary pattern above the cohort median. These curves describe variation in age at diagnosis within a case-only sample and do not estimate population incidence or disease risk

Kaplan–Meier survival curves (Fig. 1a) demonstrated that participants with higher levels of combined exposure had lower probabilities of remaining undiagnosed with pancreatic cancer at older ages than those with fewer or no exposures. The separation of curves was most pronounced for participants with two or three exposures who exhibited earlier declines in the probability of not yet being diagnosed.

After adjustment for sex, education, family history, physical activity, and chemical exposure (Fig. 1b), Cox proportional hazards models showed consistently steeper declines in the probability of not yet being diagnosed for participants with two or three exposures relative to those with no or a single exposure, indicating that multiple exposures were associated with earlier age at diagnosis.

The regression results (Table 2) further supported these findings. In Model 1, female patients were diagnosed earlier than males (HR = 1.80, 95% CI: 1.14–2.85, *p* = 0.012), and this association became stronger after adjustment in Model 2 (HR = 2.28, 95% CI: 1.45–3.56, *p* < 0.001). Higher educational attainment was consistently associated with earlier diagnosis in both models (per year increase: HR = 1.11, 95% CI: 1.07–1.16; *p* < 0.001).

**Table 2:** Association between risk factors and age at pancreatic cancer diagnosis among cases (*N* = 134). HRs estimate earlier age at diagnosis, not incident disease risk

Variable	Model 1		Model 2	
	HR (95% CI)	*p* value	HR (95% CI)	*p* value
Sex				
Male	Reference		Reference	
Female	1.80 (1.14–2.85)	0.012	2.28 (1.45–3.56)	< 0.001
**Education (years)**	1.11 (1.07–1.16)	< 0.001	1.11 (1.07–1.16)	< 0.001
**Family history of pancreatic cancer**				
No	Reference		Reference	
Yes	1.28 (0.51–3.22)	0.606	1.30 (0.51–3.33)	0.581
**Average exercise intensity** ^**a**^	0.62 (0.34–1.12)	0.114	0.54 (0.30–0.95)	0.033
**Cigarette smoking**				
Never	Reference		-	-
Former	0.98 (0.58–1.68)	0.949	-	-
Current	2.71 (1.52–4.82)	< 0.001	-	-
**Alcohol consumption**				
Never	Reference		-	-
Former	1.67 (0.95–2.94)	0.077	-	-
Current	1.61 (0.87–2.96)	0.129	-	-
**Diet index** ^**b**^	2.19 (1.64–2.91)	< 0.001	-	-
**Chemical index** ^**c**^	0.69 (0.52–0.93)	0.016	0.86 (0.66–1.12)	0.257
**Combined exposure index** ^**d**^				
0	-	-	Reference	
1	-	-	1.79 (1.14–2.81)	0.011
2	-	-	2.97 (1.67–5.28)	< 0.001
3	-	-	6.66 (3.35–13.22)	< 0.001

Abbreviations: CI = confidence interval; HR = hazard ratio; N = number of participants; WQS = weighted quantile sum. HR > 1 indicates earlier age at diagnosis among pancreatic cancer cases and should not be interpreted as incident disease risk

^a^ Time-weighted average exercise score from age 15 to current age (0 = never, 0.5 = intermittent, 1 = consistent), averaged across observed years

^b^ Dietary index constructed using grouped weighted quantile sum (WQS) regression of ten two-year variables (Chinese medicine use and nine food items), standardized to weekly servings, percentile-normalized (0–100), higher values = greater intake

^c^ Chemical exposure index constructed using grouped WQS regression of ten variables (e.g., pesticide, metal, degreaser, incense), harmonized to annual frequency, imputed if missing, percentile-normalized (0–100), higher values = greater cumulative exposure

^d^ Combined exposure index was constructed as the sum of three binary indicators—cigarette smoking (current or former = 1, never = 0), alcohol consumption (current or former = 1, never = 0), and dietary pattern (above the cohort median = 1, at or below = 0). The index ranged from 0 to 3, with 0 = none, 1 = single, 2 = double, and 3 = triple exposure. In the Cox proportional hazards models, this index was entered as a categorical variable to estimate the association between cumulative adverse lifestyle exposures and earlier age at diagnosis among pancreatic cancer cases

Among lifestyle factors, current smoking (HR = 2.71, 95% CI: 1.52–4.82, *p* < 0.001) was significantly associated with earlier diagnosis in Model 1, whereas former smoking and alcohol consumption showed a similar direction of association but did not reach statistical significance. A higher dietary index was also linked to earlier diagnosis (HR = 2.19, 95% CI: 1.64–2.91, *p* < 0.001). Family history was not significantly associated with age at diagnosis in either model. In contrast, exercise intensity was significantly associated with age at diagnosis in Model 2, while the chemical exposure index was significant in Model 1 but not in Model 2.

When lifestyle exposures were combined into a single index (Model 2), an exposure–response gradient pattern was observed. Compared with patients with no exposure, those with single, double, and triple exposure were associated with progressively higher hazards of earlier diagnosis (HR = 1.79, 95% CI: 1.14–2.81, *p* = 0.011; HR = 2.97, 95% CI: 1.67–5.28, *p* < 0.001; and HR = 6.66, 95% CI: 3.35–13.22, *p* < 0.001, respectively).

The proportional hazards assumption was generally satisfied for the Cox models. Deviations were observed for education in both models and for the chemical exposure index in Model 2 (see Table **S1** in Additional file 4). Importantly, the cumulative exposure index, which was the primary exposure of interest, met the proportional hazards assumption, supporting the robustness of the main findings. Sensitivity analyses using reduced Cox models showed consistent associations between the combined exposure index and earlier pancreatic cancer diagnosis across all reduced models (see Table **S2** in Additional file 4). We additionally adjusted for tumor stage at diagnosis among participants with available stage information (*n* = 131). The results remained directionally consistent, and the exposure–response gradient between the combined exposure index and earlier age at diagnosis persisted after further adjustment for stage (see Table **S3** in Additional file 4).

## Discussion

In this hospital-based study in Taiwan, we examined how the cumulative burden of lifestyle exposure is associated with age at pancreatic cancer diagnosis. Our key finding is an exposure–response gradient association, with increasing adverse lifestyle exposure count associated with earlier age at diagnosis. Compared with unexposed individuals, those with one, two, and three exposures had progressively higher hazards of earlier diagnosis (HR = 1.79, 2.97, and 6.66, respectively). The increasing divergence of survival curves across exposure groups further supports an association between greater combined exposure burden and earlier age at pancreatic cancer diagnosis.

These findings align with prior evidence showing that cigarette smoking and heavy alcohol consumption independently advance pancreatic cancer onset by several years [19, 22]. Our study extends this understanding by integrating smoking, alcohol use, and adverse dietary patterns into a combined exposure index, providing a systematic and quantitative assessment of overall lifestyle-related risk. This approach is conceptually similar to the exposure-counting models described by Fukai et al. [34] and is consistent with the broader “exposome” paradigm, which emphasizes the combined impact of multiple environmental and behavioral exposures on cancer development [4, 5]. The markedly elevated hazard among individuals with three concurrent exposures suggests a potential synergistic effect in which tobacco carcinogens, alcohol-induced metabolic stress, and unhealthy dietary components may jointly contribute to biological processes relevant to earlier pancreatic cancer diagnosis [22, 35–38].

Education emerged as an independent predictor of earlier age at diagnosis (HR = 1.11, *p* < 0.001), with higher educational attainment being associated with younger age at diagnosis. Although education is a recognized social determinant of health [39], its relationship with cancer onset is complex. Lower education is often linked to higher cancer-specific mortality and later-stage diagnosis, potentially due to reduced access to screening and timely care [40, 41]. Conversely, individuals with higher education may be more attuned to bodily symptoms or more likely to seek early evaluation, which may reflect differences in detection rather than underlying biological differences. Lifestyle risk factors are also clustered by socioeconomic status [40], which may partially explain the association between education and the combined exposure index observed in our analysis. In addition, differences in health awareness or health-seeking behavior across educational groups may influence the timing of diagnosis. Because this study was conducted in a hospital-based population, differences in referral patterns or access to medical care across socioeconomic groups could also contribute to the observed association.

Our grouped WQS-derived dietary index identified high-risk dietary components associated with early diagnosis. Fried chicken, French fries, and sugar-sweetened beverages were the strongest contributors, consistent with their high contents of advanced glycation end products (AGEs) and refined sugars. These compounds promote systemic inflammation, oxidative stress, and insulin resistance, all of which are implicated in pancreatic tumorigenesis [42–44]. These metabolic and inflammatory pathways may interact with tobacco and alcohol exposure [45–47], reinforcing the notion that combined lifestyle and dietary risks jointly influence disease onset.

We also observed that female patients were diagnosed significantly earlier than male patients (HR = 2.28, 95% CI: 1.45–3.56), independent of combined exposure burden, education, family history, and chemical exposures. This finding contrasts with descriptive statistics showing no sex difference in the mean age at diagnosis and with the higher prevalence of smoking and alcohol use in men, suggesting that factors related to sex may be associated with differences in the timing of diagnosis. However, additional analyses indicated that stage at diagnosis differed by sex, with men more likely to present with more advanced disease. This suggests that differences in stage at presentation may partly contribute to the observed earlier age at diagnosis among women. Similar patterns have been reported in pancreatic neuroendocrine tumors [48], pointing to potential biological mechanisms. Estrogen and other sex hormones regulate pathways relevant to tumor initiation and progression [49, 50], potentially enhancing the susceptibility of precursor lesions to malignant transformation [51]. Interactions between hormonal regulation and lifestyle exposures may contribute to accelerated carcinogenesis in women, warranting further mechanistic investigations.

Interestingly, family history was not significantly associated with age at diagnosis in either model. The chemical exposure index was associated with age at diagnosis in Model 1 but not in Model 2. Although these factors influence overall pancreatic cancer risk [52, 53], their associations with age at diagnosis may be subtle or require larger cohorts to detect. It is also possible that the combined lifestyle exposure index in Model 2 captured overlapping effects, attenuating the independent contribution of individual factors.

The observed exposure–response gradient association is biologically plausible, as smoking, alcohol consumption, and unhealthy diets are linked to pathways relevant to pancreatic tumor development. Tobacco-specific nitrosamines and alcohol-derived acetaldehyde have been associated with KRAS mutations, oxidative stress, and chronic inflammation, whereas high-AGE and high-sugar diets may exacerbate metabolic dysfunction and insulin resistance [37, 38, 42–47]. These combined insults may contribute to genomic instability, pancreatic stellate cell activation, and a pro-tumor inflammatory microenvironment. However, because the combined exposure index was discrete and data-derived, these mechanisms should be viewed as possible explanations for the observed timing pattern rather than proof of a calibrated biological dose gradient or accelerated carcinogenesis.

This study has several strengths, including the development of a structured combined exposure index, application of grouped WQS regression to quantify exposure mixtures, and use of Cox time-to-event models tailored to onset-focused questions. By integrating lifestyle, dietary, occupational, and socioeconomic factors, this study provides a comprehensive evaluation of how combined exposures are associated with the timing of pancreatic cancer diagnosis.

### Limitations of the Study

Several limitations should be considered when interpreting these findings. First, the hospital-based, case-only design may introduce selection bias and referral bias because patients treated at a tertiary referral center may differ from all patients with pancreatic cancer in Taiwan. Conditioning on having pancreatic cancer may also introduce collider bias, which could generate or distort associations between exposures and age at diagnosis. The direction of this bias is difficult to predict and could either exaggerate or attenuate the observed associations.

Second, this case-only study cannot assess pancreatic cancer risk or incidence. The associations reported here reflect variation in age at diagnosis among individuals who already had pancreatic cancer. Age at diagnosis may be influenced by detection patterns, healthcare access, symptom awareness, referral pathways, and stage at presentation, rather than only by biological disease onset. Although adjustment for tumor stage yielded directionally consistent results, residual differences in detection and referral could still affect the magnitude of the estimates.

Third, exposure measurement relied partly on retrospective self-report, especially for diet, alcohol consumption, and occupational or environmental chemical exposures. Recall error and misclassification may have occurred. If measurement error was largely nondifferential, it may have attenuated associations for individual exposures; however, age-related differences in recall or reporting could also bias estimates in either direction. Dichotomizing continuous or ordinal exposures, including the dietary index and alcohol consumption, improved interpretability but reduced granularity, may have introduced exposure misclassification, and could make the observed exposure-count pattern appear more or less regular than the underlying relationships.

Fourth, residual confounding remains possible because information on body mass index, detailed healthcare access, and other metabolic or clinical factors was unavailable. Because these factors may be related to both lifestyle exposures and timing of diagnosis, unmeasured confounding could either partially explain or mask the observed associations. Education was included as a proxy for socioeconomic status, but it may not fully capture access to care, health literacy, or health-seeking behavior.

Fifth, the statistical models should be interpreted cautiously. Cox proportional hazards models were used with age at diagnosis as the time scale and no censoring because all participants had pancreatic cancer. Therefore, HRs should be read as relative differences in age at diagnosis among cases, not as incident risk estimates. The modest sample size, grouped WQS regression, multiple covariates, and data-driven construction of the combined exposure index may increase the potential for overfitting, instability, and inflation of effect estimates. Although confidence intervals were reported, precision was limited for higher exposure categories, especially triple exposure (HR = 6.66, 95% CI: 3.35–13.22), indicating uncertainty around the magnitude of the association despite statistical significance.

Finally, generalizability may be limited. The study was conducted at a single tertiary referral hospital in Taiwan, and lifestyle patterns, healthcare systems, referral practices, and diagnostic pathways may differ across populations. Taken together, these limitations suggest that the results are best interpreted as exploratory and hypothesis-generating. Some biases, such as data-driven index construction and referral patterns, may overestimate the strength or regularity of the association, whereas nondifferential exposure misclassification may attenuate individual exposure associations.

## Conclusions

Our findings suggest that a higher count of adverse lifestyle exposures is associated with earlier age at pancreatic cancer diagnosis among cases. Reducing cigarette smoking, excessive alcohol consumption, and unhealthy dietary patterns may help reduce the burden of modifiable risk factors associated with earlier pancreatic cancer diagnosis. Although the combined exposure index is not intended for direct clinical application and does not estimate disease incidence, it may help identify individuals with a higher burden of modifiable risk factors who could benefit from increased clinical awareness and earlier evaluation. Future studies should investigate underlying biological mechanisms, assess exposure synergy in larger cohorts, and validate these findings across diverse populations.

## Supplementary Information

Below is the link to the electronic supplementary material.

Supplementary Material 1

Supplementary Material 2

Supplementary Material 3

Supplementary Material 4

## Data Availability

The data supporting the findings of this study are available on request from the corresponding author. These data are not publicly available as they contain information that could compromise the privacy of research participants. Access to the data must be further approved by the Research Ethics Committee III of China Medical University and Hospital.

## Abbreviations

AGEs: Advanced glycation end products
CI: Confidence interval
CMUH: China Medical University Hospital
HR: Hazard ratio
ICD-10: International Classification of Diseases, 10th Revision
IRB: Institutional Review Board
SD: Standard deviation
STROBE: Strengthening the Reporting of Observational Studies in Epidemiology
WQS: Weighted quantile sum
