# Supplementary material for: Association between a combined lifestyle exposure index and age at pancreatic cancer diagnosis: a hospital-based retrospective study
Source: BMC Gastroenterol. 2026 Sep 10;26:709. doi: 10.1186/s12876-026-05297-0 (PMC13718021; doi:10.1186/s12876-026-05297-0)
Supplement: Supplementary file 1 [file 12876_2026_5297_MOESM2_ESM.pdf]

# STROBE Participant Flow Chart

*Combined lifestyle exposure index study:*
*complete-case analytic dataset*

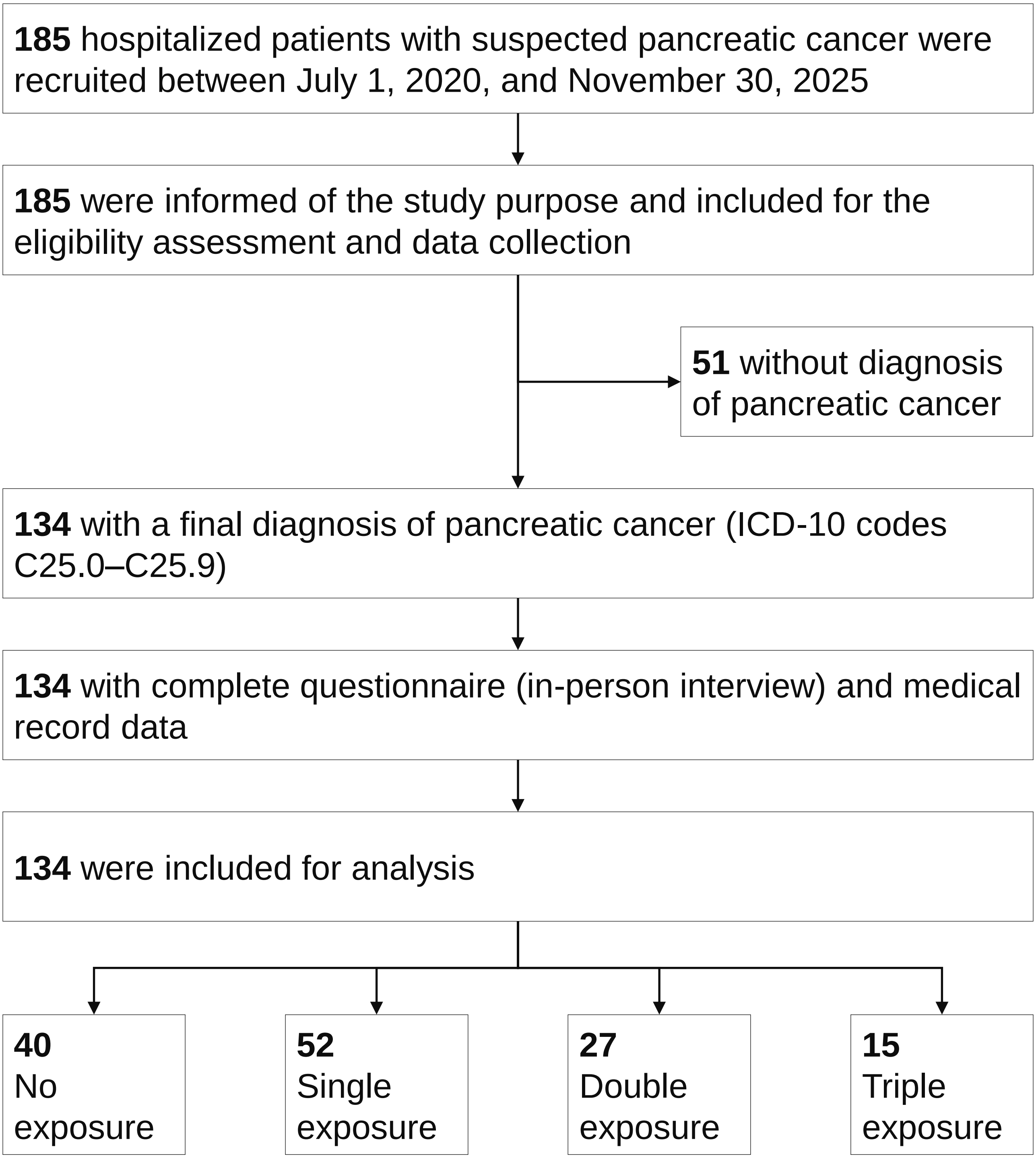


**Note:** Counts reflect the complete-case analytic dataset described in the revised manuscript. The combined exposure index was categorized as 0 = none, 1 = single exposure, 2 = double exposure, and 3 = triple exposure. All 134 confirmed pancreatic cancer cases had complete questionnaire data, so no participants were excluded for incomplete questionnaire data.
