# Supplementary material for: Association between a combined lifestyle exposure index and age at pancreatic cancer diagnosis: a hospital-based retrospective study
Source: BMC Gastroenterol. 2026 Sep 10;26:709. doi: 10.1186/s12876-026-05297-0 (PMC13718021; doi:10.1186/s12876-026-05297-0)
Supplement: Supplementary file 2 [file 12876_2026_5297_MOESM3_ESM.pdf]

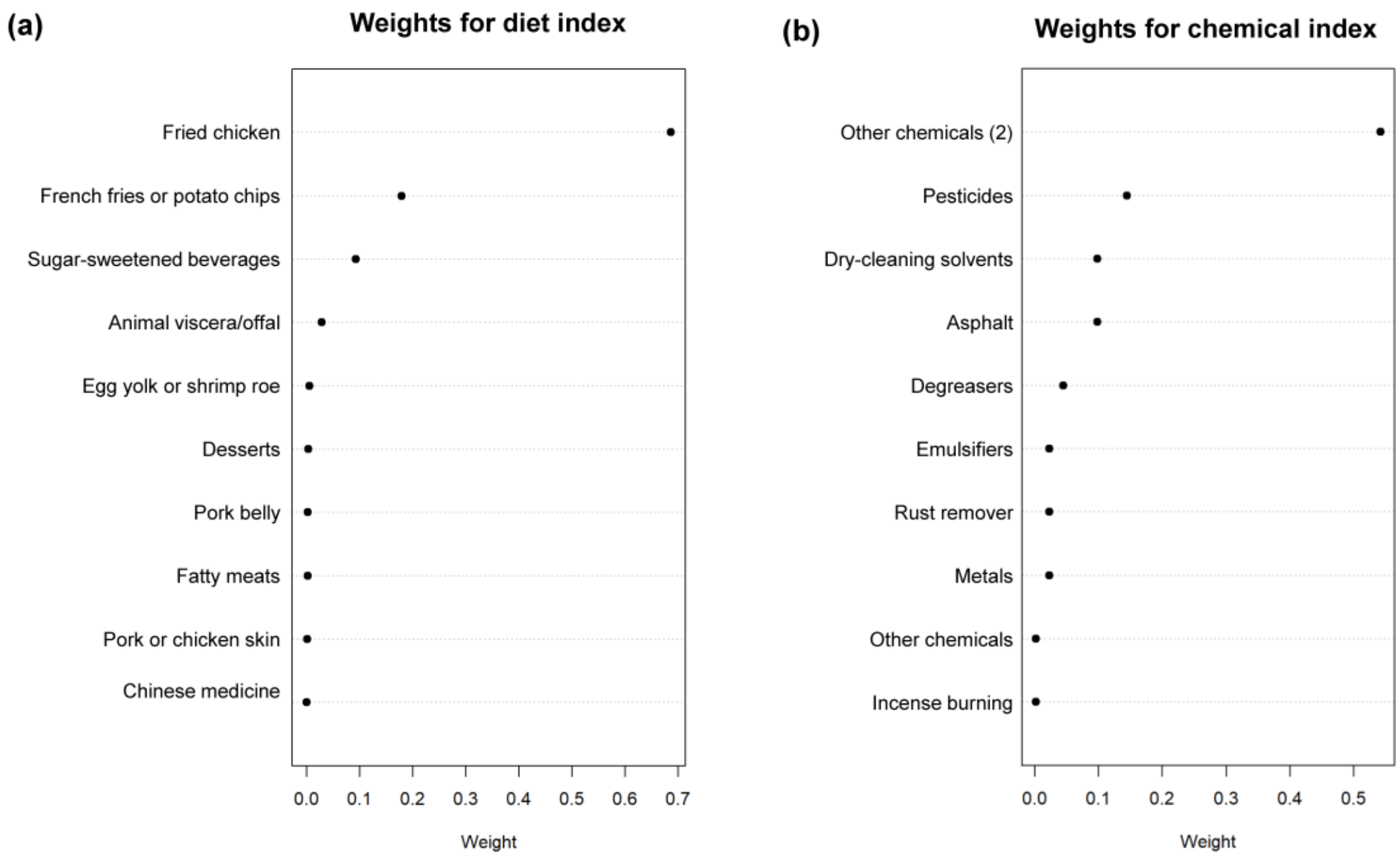


**Figure S1 Relative weighted contributions of individual dietary (a) and chemical/occupational (b) components to the weighted quantile sum indices**

Dietary components, in order of display, included fried chicken, French fries or potato chips, sugar-sweetened beverages, animal viscera/offal, egg yolk or shrimp roe, desserts, pork belly, fatty meats, pork or chicken skin, and Chinese medicine (per week). Chemical/occupational exposures included other chemicals (2), pesticides, dry-cleaning solvents, asphalt, degreasers, emulsifiers, rust remover, metals, other chemicals, and incense burning.
