# Supplementary material for: Association between a combined lifestyle exposure index and age at pancreatic cancer diagnosis: a hospital-based retrospective study
Source: BMC Gastroenterol. 2026 Sep 10;26:709. doi: 10.1186/s12876-026-05297-0 (PMC13718021; doi:10.1186/s12876-026-05297-0)
Supplement: Supplementary file 3 [file 12876_2026_5297_MOESM4_ESM.pdf]

Table S1. Tests of proportional hazards assumption using Schoenfeld residual-based diagnostics

Variable	Model 1 p value	Model 2 p value
**Sex (Female vs. Male)**	0.880	0.742
**Education (years)**	0.002	0.006
**Family history of pancreatic cancer (Yes vs. No)**	0.226	0.316
**Average exercise intensity** [a]	0.611	0.791
**Cigarette smoking**		
Former vs. Never	0.355	—
Current vs. Never	0.414	—
**Alcohol consumption**		
Former vs. Never	0.357	—
Current vs. Never	0.729	—
**Dietary index** [b]	0.731	—
**Chemical index** [c]	0.094	0.020
**Combined exposure index** [d]		
1 vs. 0	—	0.295
2 vs. 0	—	0.511
3 vs. 0	—	0.453

Proportional hazards assumption was evaluated using Schoenfeld residual–based diagnostics with 5,000 resampling iterations and a fixed random seed. P-values < 0.05 indicate potential violation of the proportional hazards assumption. "—" indicates that the variable was not included in the corresponding model.

[a] Time-weighted average exercise score from age 15 to current age (0 = never, 0.5 = intermittent, 1 = consistent), averaged across observed years

[b] Dietary index constructed using grouped WQS regression of ten two-year variables (Chinese medicine use and nine food items), standardized to weekly servings, percentile-normalized (0–100), higher values = greater intake.

[c] Chemical exposure index constructed using grouped WQS regression of ten variables (e.g., pesticide, metal, degreaser, incense), harmonized to annual frequency, imputed if missing, percentile-normalized (0–100), higher values = greater cumulative exposure.

[d] Combined exposure index was constructed as the sum of three binary indicators—cigarette smoking (current or former = 1, never = 0), alcohol consumption (current or former = 1, never = 0), and dietary pattern (above the cohort median = 1, at or below = 0). The index ranged from 0 to 3, with 0 = none, 1 = single, 2 = double, and 3 = triple exposure. In the Cox proportional hazards models, this index was entered as a categorical variable to estimate the association between cumulative adverse lifestyle exposures and earlier age at diagnosis among pancreatic cancer cases.

Table S2. Sensitivity analyses for the association between the combined exposure index and age at pancreatic cancer diagnosis

**Model**	**HR (95% CI) for combined exposure index (reference: none)**		
	**1 (single exposure)**	**2 (double exposure)**	**3 (triple exposure)**
**Main Model (Model 2)**	1.79 (1.14–2.81)	2.97 (1.67–5.28)	6.66 (3.35–13.22)
**Remove “Sex” variable**	1.61 (1.03–2.51)	1.92 (1.15–3.21)	4.14 (2.21–7.78)
**Remove “Education” variable**	1.58 (1.01–2.47)	2.36 (1.34–4.14)	5.29 (2.65–10.54)
**Remove “Family history of pancreatic cancer” variable**	1.81 (1.16–2.84)	3.01 (1.70–5.34)	6.57 (3.32–13.02)
**Remove “Average exercise intensity” variable**	1.91 (1.22–3.00)	3.32 (1.87–5.87)	6.72 (3.37–13.37)
**Remove “Chemical index” variable**	1.72 (1.10–2.69)	3.07 (1.73–5.45)	6.58 (3.31–13.07)

HR = hazard ratio; CI = confidence interval.
The combined exposure index was categorized as 0 (none), 1 (single exposure), 2 (double exposure), and 3 (triple exposure).
Hazard ratios were estimated using Cox proportional hazards models with age at pancreatic cancer diagnosis as the time scale.

Table S3. Association between risk factors and age at diagnosis with pancreatic cancer, adjusted for tumor stage at diagnosis (N = 131)

Variable	Model 1		Model 2	
	HR (95% CI)	p value	HR (95% CI)	p value
**Sex**				
Male	Reference		Reference	
Female	1.89 (1.17–3.06)	0.010	2.59 (1.62–4.15)	<0.001
**Education (years)**	1.12 (1.07–1.17)	<0.001	1.12 (1.08–1.17)	<0.001
**Family history of pancreatic cancer**				
No	Reference		Reference	
Yes	1.18 (0.46–3.01)	0.727	1.11 (0.43–2.87)	0.827
**Average exercise intensity** [a]	0.59 (0.32–1.09)	0.090	0.53 (0.29–0.97)	0.041
**Cigarette smoking**				
Never	Reference		-	-
Former	1.08 (0.61–1.91)	0.785	-	-
Current	2.65 (1.45–4.85)	0.002	-	-
**Alcohol consumption**				
Never	Reference		-	-
Former	1.51 (0.84–2.74)	0.172	-	-
Current	1.70 (0.89–3.24)	0.109	-	-
**Diet index** [b]	2.29 (1.71–3.07)	<0.001	-	-
**Chemical index** [c]	0.69 (0.51–0.94)	0.018	0.87 (0.67–1.13)	0.300
**Combined exposure index** [d]				
0	-	-	Reference	
1	-	-	2.17 (1.34–3.51)	0.002
2	-	-	4.18 (2.22–7.86)	<0.001
3	-	-	8.48 (4.03–17.88)	<0.001
**Tumor stage**				
I	-	-	Reference	
II	1.61 (0.79–3.27)	0.191	1.62 (0.81–3.25)	0.175
III	1.44 (0.75–2.80)	0.276	1.81 (0.93–3.54)	0.082
IV	1.89 (1.03–3.48)	0.040	2.31 (1.25–4.28)	0.008

HR = hazard ratio; CI = confidence interval.

The combined exposure index was categorized as 0 (none), 1 (single exposure), 2 (double exposure), and 3 (triple exposure).

Hazard ratios were estimated using Cox proportional hazards models with age at pancreatic cancer diagnosis as the time scale.
