# Supplementary material for: Association between a combined lifestyle exposure index and age at pancreatic cancer diagnosis: a hospital-based retrospective study
Source: BMC Gastroenterol. 2026 Sep 10;26:709. doi: 10.1186/s12876-026-05297-0 (PMC13718021; doi:10.1186/s12876-026-05297-0)
Supplement: Supplementary file 4 [file 12876_2026_5297_MOESM1_ESM.docx]

**STROBE Statement—checklist of items that should be included in reports of observational studies**

|  | Item No. | Recommendation | Page  No. | Relevant text from manuscript |
| --- | --- | --- | --- | --- |
| **Title and abstract** | 1 | (*a*) Indicate the study’s design with a commonly used term in the title or the abstract | Pages 1–2 | Title revised to state the study design: hospital-based retrospective study. Abstract Methods states a hospital-based retrospective case-only study. |
|  |  | (*b*) Provide in the abstract an informative and balanced summary of what was done and what was found | Pages 2–3 | Structured abstract summarizes background, methods, results with HRs and 95% CIs, conclusions, trial registration, and keywords. |
| Introduction | | | |  |
| Background/rationale | 2 | Explain the scientific background and rationale for the investigation being reported | Pages 3–4 | Background explains increasing younger-onset cancer burden, pancreatic cancer context, and rationale for evaluating cumulative exposures. |
| Objectives | 3 | State specific objectives, including any prespecified hypotheses | Page 4 | Objective states investigation of whether accumulation of adverse exposures is associated with younger age at pancreatic cancer diagnosis. |
| Methods | | | |  |
| Study design | 4 | Present key elements of study design early in the paper | Page 4 | Key design elements presented early: hospital-based retrospective case-only observational study at CMUH. |
| Setting | 5 | Describe the setting, locations, and relevant dates, including periods of recruitment, exposure, follow-up, and data collection | Page 5 | Setting, hospital, departments, recruitment dates, and data collection period are described. |
| Participants | 6 | (*a*) *Cohort study*—Give the eligibility criteria, and the sources and methods of selection of participants. Describe methods of follow-up  *Case-control study*—Give the eligibility criteria, and the sources and methods of case ascertainment and control selection. Give the rationale for the choice of cases and controls  *Cross-sectional study*—Give the eligibility criteria, and the sources and methods of selection of participants | Page 5 | Eligibility criteria, ICD-10 diagnosis codes, recruitment sources, consent, and exclusion of incomplete questionnaire data are described. |
|  |  | (*b*) *Cohort study*—For matched studies, give matching criteria and number of exposed and unexposed  *Case-control study*—For matched studies, give matching criteria and the number of controls per case | NA | No matched cohort or matched case-control design was used. |
| Variables | 7 | Clearly define all outcomes, exposures, predictors, potential confounders, and effect modifiers. Give diagnostic criteria, if applicable | Pages 5–8 | Outcome, exposures, covariates, diagnostic criteria, and exposure index definitions are specified. |
| Data sources/ measurement | 8* | For each variable of interest, give sources of data and details of methods of assessment (measurement). Describe comparability of assessment methods if there is more than one group | Pages 5–7 | Sources and measurement methods are described: structured interviews and medical record review. |
| Bias | 9 | Describe any efforts to address potential sources of bias | Pages 6, 16–18 | Bias-reduction procedures and remaining risks of recall, measurement, selection, referral, and collider bias are described. |
| Study size | 10 | Explain how the study size was arrived at | Page 5 | Study size determined by eligible, consenting patients with complete questionnaire data during the recruitment period; no formal sample size calculation. |
| Quantitative variables | 11 | Explain how quantitative variables were handled in the analyses. If applicable, describe which groupings were chosen and why | Pages 6–10 | Categorization and handling of education, smoking, alcohol, exercise, diet, chemical exposure, and combined exposure index are described. |
| Statistical methods | 12 | (a) Describe all statistical methods, including those used to control for confounding | Pages 8–10 | Descriptive tests, WQS regression, Cox models, covariate adjustment, proportional hazards diagnostics, and stage sensitivity analyses are described. |
|  |  | (b) Describe any methods used to examine subgroups and interactions | Page 10 | No subgroup or interaction analyses were performed; a tumor stage-adjusted sensitivity analysis was conducted. |
|  |  | (c) Explain how missing data were addressed | Pages 5, 7 | Participants with incomplete questionnaire data were excluded; missing diet/chemical component values were imputed before percentile normalization. |
|  |  | (d) Cohort study—If applicable, explain how loss to follow-up was addressed  Case-control study—If applicable, explain how matching of cases and controls was addressed  Cross-sectional study—If applicable, describe analytical methods taking account of sampling strategy | Page 8 | No loss to follow-up or censoring occurred because all participants were pancreatic cancer cases and age at diagnosis was observed. |
|  |  | (e) Describe any sensitivity analyses | Page 10 | Reduced Cox models and tumor stage-adjusted sensitivity analyses are described and reported in Additional file 4 (Tables S2 and S3). |
| Results |  |  |  |  |
| Participants | 13* | (a) Report numbers of individuals at each stage of study—eg numbers potentially eligible, examined for eligibility, confirmed eligible, included in the study, completing follow-up, and analysed | Page 10 | Final analytic sample included 134 patients; flow chart reports inclusion and final exposure-index group counts. |
|  |  | (b) Give reasons for non-participation at each stage | Pages 5, 10 | Incomplete questionnaire data were exclusionary; no additional exclusions were recorded in the complete-case analytic file. |
|  |  | (c) Consider use of a flow diagram | Page 10 | Editable STROBE participant flow chart supplied as Additional file 2. |
| Descriptive data | 14* | (a) Give characteristics of study participants (eg demographic, clinical, social) and information on exposures and potential confounders | Pages 10–11, 28–29 | Participant demographic, clinical, social, lifestyle, dietary, chemical, and combined exposure characteristics are reported. |
|  |  | (b) Indicate number of participants with missing data for each variable of interest | Pages 5, 13 | Complete questionnaire data were required; tumor stage sensitivity analysis reports available stage data (n = 131). |
|  |  | (c) Cohort study—Summarise follow-up time (eg, average and total amount) | Page 8 | No follow-up time was accrued; the outcome was age at pancreatic cancer diagnosis among cases. |
| Outcome data | 15* | Cohort study—Report numbers of outcome events or summary measures over time | Pages 10–11 | Outcome age at diagnosis is summarized; all participants had pancreatic cancer diagnosis. |
|  |  | Case-control study—Report numbers in each exposure category, or summary measures of exposure | NA | No controls were included. |
|  |  | Cross-sectional study—Report numbers of outcome events or summary measures | NA | This was not a cross-sectional prevalence study. |
| Main results | 16 | (a) Give unadjusted estimates and, if applicable, confounder-adjusted estimates and their precision (eg, 95% confidence interval). Make clear which confounders were adjusted for and why they were included | Pages 11–12, 30 | Descriptive unadjusted comparisons of age at diagnosis (Table 1) and confounder-adjusted Cox model estimates with 95% CIs (Table 2) are reported; the covariates adjusted for are specified. |
|  |  | (b) Report category boundaries when continuous variables were categorized | Pages 9–10 | Category boundaries for combined exposure index and dichotomized variables are reported. |
|  |  | (c) If relevant, consider translating estimates of relative risk into absolute risk for a meaningful time period | Pages 8, 17–18 | Relative estimates are timing estimates among cases, not incident risk estimates over a meaningful time period. |
| Other analyses | 17 | Report other analyses done—eg analyses of subgroups and interactions, and sensitivity analyses | Pages 11, 13 | WQS component weights (Figure S1, Additional file 3) and proportional hazards diagnostics, reduced-model sensitivity analyses, and stage-adjusted analyses (Tables S1–S3, Additional file 4) are reported. |
| **Discussion** | | | | |
| Key results | 18 | Summarise key results with reference to study objectives | Page 13 | Key results summarized with reference to study objectives. |
| Limitations | 19 | Discuss limitations of the study, taking into account sources of potential bias or imprecision. Discuss both direction and magnitude of any potential bias | Pages 16–18 | Limitations address selection/referral bias, collider bias, measurement error, residual confounding, precision, overfitting, dichotomization, and generalizability, including likely direction of bias where feasible. |
| Interpretation | 20 | Give a cautious overall interpretation of results considering objectives, limitations, multiplicity of analyses, results from similar studies, and other relevant evidence | Pages 13–18 | Interpretation is cautious and frames results as age-at-diagnosis associations among cases rather than disease risk or causality. |
| Generalisability | 21 | Discuss the generalisability (external validity) of the study results | Page 18 | Generalizability beyond a tertiary referral population in Taiwan is discussed. |
| **Other information** | | | | |
| Funding | 22 | Give the source of funding and the role of the funders for the present study and, if applicable, for the original study on which the present article is based | Page 20 | Source of funding (National Science and Technology Council and China Medical University, Taiwan) and the statement that the funders had no role in study design, data collection, analysis, interpretation, writing, or the decision to publish are reported. |

**Note:** An Explanation and Elaboration article discusses each checklist item and gives methodological background and published examples of transparent reporting. The STROBE checklist is best used in conjunction with this article (freely available on the Web sites of PLoS Medicine at http://www.plosmedicine.org/, Annals of Internal Medicine at http://www.annals.org/, and Epidemiology at http://www.epidem.com/). Information on the STROBE Initiative is available at www.strobe-statement.org.
